# The effect of proniosomal hydroxytyrosol enriched extract added during pre- and post-fermentation of yoghurt production

**DOI:** 10.3389/fnut.2026.1829669

**Published:** 2026-06-05

**Authors:** Büşra Alper, Ceren Ilgaz, Haşim Kelebek, Pınar Kadiroglu

**Affiliations:** Department of Food Engineering, Adana Alparslan Türkes Science and Technology University, Adana, Türkiye

**Keywords:** encapsulation, hydroxytyrosol, olive leaf, proniosome, yoghurt

## Abstract

Olive leaf polyphenols are bioactive components that known for their health-protective properties. It is well established that oleuropein and its main hydrolysis product, hydroxytyrosol, possess some biologically active properties, including antioxidant and antimicrobial activities. However, these compounds may lose their beneficial properties in unprotected form, resulting in undesirable flavors in food products. In the present study, nanocapsules containing the lyophilised olive leaf brine extract (OLBE) in the form of proniosomes, were added to yoghurt (post-fermentation) and milk (pre-fermentation) samples at varying concentrations. The yoghurt samples were then analyzed for physicochemical properties, antioxidant capacity, microbiological properties, textural properties, bioaccessibility of bioactive compounds by the *in vitro* digestive system method, volatile compound analysis, and sensory properties. The effect of fermentation was similar in both methods and the best results were obtained with the addition of 0.5 g proniosome. The incorporation of nanocapsules into yoghurt resulted in an enhancement of antioxidant activity during both fermentation processes. No significant change was observed in physicochemical and sensory properties, but the syneresis rate decreased compared to yoghurt containing unencapsulated extract. At the end of simulated *in vitro* digestion, the antioxidant activities determined by DPPH and ABTS methods of yoghurt with post-fermentation additions were significantly higher than those with pre-fermentation additions. The present findings emphasize the potential of fermented olive leaf extract-loaded proniosomes as a functionally enhanced yoghurt additive. This additive has the capacity to fortify yoghurt while concurrently enhancing its nutritional value and quality.

## Introduction

In recent years, natural extracts and by-products rich in bioactive compounds have attracted great interest from both the public and scientists. With this interest, there is a need to meet the increasing demand for natural products and compounds and to produce new functional foods with important health benefits.

Some of the by-products from the collection and industrial processing of agricultural products contain various bioactive compounds (1). Olive and olive oil production wastes and by-products have been proposed as functional food product additives (2). Among these compounds, oleuropein, beyond having the potential to be added to food formulations, has additional benefits with the formation of hydroxytyrosol because of successful hydrolysis (3). Hydroxytyrosol (HT) is prevalent in plants of the *Oleaceae* family and serves as a natural antioxidant with amphiphilic properties. It exhibits good antimicrobial, anti-inflammatory, anticancer and antioxidant properties demonstrating potential in medical applications (4).

The culinary use of olive leaf extract and other natural polyphenols is constrained by their bitter taste and variability in the food matrix. Moreover, the bioavailability of polyphenols is quite poor in unprotected form. Encapsulation technology is an effective method to overcome these challenges and mask the unpleasant taste as well as maintain bioactive constituents (5). Proniosomes are non-ionic surfactant vesicles available in two forms: dry granular proniosomes and liquid crystalline proniosomes (proniosomal gel). As carriers, proniosomes act as reservoirs for the release of encapsulated bioactives through attainment of their characteristics by selecting appropriate surfactants for the proniosome formulation (6). Proniosomes are vesicular systems that are the dry form of niosomes and have been developed to overcome some of the stability limitations of their liquid counterparts. Proniosomes are free-flowing, dry powders of a surfactant-coated carrier that can be hydrated in aqueous food matrices such as milk, yoghurt or functional drinks to form niosomes (7). Furthermore, proniosomal powders have multiple advantages compared to their liquid counterparts, including ease of dosing and handling as well as reduced risk of microbial contamination (8).

Yoghurt is among the most prevalent and widely consumed products in the field of health and food sectors globally. Therefore, there are several researches on yoghurt fortification using different plant extracts such as cinnamon leaf (9), pomegranate peel (10), mango peel (10), grape and callus (12), tea (13) and different edible flower (14) extracts. In this study, nanoparticles were produced applying the proniosome method and the lyophilised extract obtained from fermented olive leaf brine enriched with hydroxytyrosol was encapsulated by these nanocarriers. Subsequently, this proniosomal powder was added to yoghurt and milk during both pre and post fermentation and then examined for its physicochemical properties, antioxidant capacity, microbiological properties, textural properties, digestibility of bioactive compounds by *in vitro* digestive tract method, volatile compound analysis and sensory properties in order to determine the possibility of producing a functional food product.

## Materials and methods

### Preparation of fermented olive leaf brine extract

The olive leaves were collected from the Gemlik variety of olive, which is cultivated in the Mediterranean region of Türkiye, and fermented olive leaf brine extracts (OLBE) were obtained through ultrasonic extraction conditions, which have been optimized and previously described in a previous study (15).

### Proniosomal powder production

The proniosomal formulation used in this study was previously optimized and comprehensively characterized (16). Proniosome production was carried out using the slurry method. Briefly, a mixture consisting of a 1:1 molar ratio of surfactant/dodecanol and the lyophilised form (200 mg) of hydroxytyrosol-enriched fermented olive leaf brine extract was dissolved in 10 mL of ethanol and transferred to a round-bottom flask, followed by the addition of maltodextrin as a carrier material. Under optimized process conditions (35 °C and 50 rpm), dry granular proniosomes were obtained after solvent evaporation using a rotary evaporator. The proniosome powder was kept at ambient temperature in the dark overnight to ensure complete drying. Also, the selected formulation (35 °C, 50 rpm) demonstrated high encapsulation efficiency (81.20%), particle size (~188–248 nm), a low polydispersity index (~0.24), and a negative zeta potential (~−30 mV), indicating its suitability as a stable delivery system (16).

### Preparation of yoghurt samples

For the post fermentation study, 1 L of fresh pasteurized milk was heat-treated to 45 °C and 1 g (0.7% w/v) yoghurt culture containing *Lactobacillus delbrueckii* subsp. *bulgaricus* and *Streptococcus thermophilus* (0.7% w/v) were inoculated into the milk and subsequently mixed. The inoculated 1 L of milk was transferred evenly into 210 cc jars with a volume of 150 mL and incubated at 45 °C and the incubation was terminated when the pH reached 4.6. At the end of incubation, the samples were kept at room temperature until its temperature was reduced to 24 °C and each yoghurt sample was stored at 4 °C for 1 day. Then, fermented milk product (yoghurt) containing various concentrations of proniosomal OLBE and OLBE was produced by adding proniosomal OLBE (0.1, 0.2, 0.3, 0.4 and 0.5 g) and free OLBE (16, 33, 50, 66 and 83 mg) to the prepared yoghurt. Plain yoghurt sample without proniosomal OLBE and free extract was defined as control. The produced yoghurt samples were stirred for 30 s to break the clot and the experiments were carried out.

For the pre-fermentation study, to prepare yoghurt enriched with both OLBE and proniosomal OLBE, 150 mL of 1 L of fresh pasteurized milk was separated and heat-treated to 80 °C and 150 mL of milk was transferred to 5 different jars of 210 cc volume as 30 mL. Proniosomal OLBE (0.1, 0.2, 0.3, 0.4 and 0.5 g) and free OLBE (16, 33, 50, 66 and 83 mg) were added to the 80 °C milk and mixed thoroughly to produce different concentrations of fermented milk products. A plain yoghurt sample without PHE and free extract was defined as control. The remaining milk was heat treated to 45 °C and 1 g (0.7% w/v) yoghurt starter culture containing *Lactobacillus delbrueckii* subsp. *bulgaricus* and *Streptococcus thermophilus* was inoculated into the milk and mixed. The inoculated milk was added to the containers with proniosomes cooled to 45 °C and mixed gently and incubated at 45 °C until the pH reached 4.6. At the end of incubation, the temperature of the samples was reduced to room temperature (24 °C) and each yoghurt sample was stored at 4 °C for 1 day. The yoghurt samples were mixed for 30 s to break the clot and the experiments were carried out.

### Determination of antioxidant activity by DPPH and ABTS method

#### Preparation of samples

A total of 2.5 mL distilled water was added to 10 g yoghurt and mixed. Then 1 M HCl was added and pH was adjusted to 4. The yoghurt was incubated at 45 °C for 10 min and then centrifuged at 7500 rpm for 30 min at 4 °C. The supernatant was separated and adjusted to pH 7 by adding NaOH. Centrifugation was performed again at 7500 rpm for 30 min at 4 °C and the supernatant was separated (17).

#### Determination of antioxidant capacity

Antioxidant activity measurements were determined by measuring the neutralizing activities of ABTS (2,2′-azino-bis(3-ethylbenzothiazoline-6-sulfonic acid) and DPPH (2,2-diphenyl-1-(2,4,6-trinitrophenyl) hydrazyl) radicals and the absorption capacity of oxygen radicals. 250 μL of the sample solution prepared in the “Preparation of samples” section was added to 3 mL ethanolic solution of DPPH (60 μM) and mixed vigorously. Then, it was kept at ambient temperature for 5 min and the absorbance at 517 nm wavelength was recorded (18). The initial absorbance value of DPPH solution was adjusted to 0.700 ± 0.020 using 60 μM methanol.

For the ABTS method, the binding capacity of ABTS free radicals was determined at 734 nm as defined by Re et al. (19) by measuring the change in the absorbance value of the reaction of 0.1 mL sample solution and 3.9 mL 7 mmol/L ABTS radical solution prepared in 2.5 mmol/L potassium persulfate (AgilentCarry 60, China). The initial absorbance value of the ABTS radical solution was adjusted to 0.700 ± 0.020 using 75 mmol/L phosphate salt buffer (containing 150 mmol/L NaCl), pH 7.4. The results were expressed as antioxidant capacity equivalent to trolox (TEAC/mg).

#### Determination of syneresis (water release) property

10 g of yoghurt was taken into a centrifuge tube and centrifuged at 2500 rpm for 10 min at 4 °C. After centrifugation, the supernatant was taken and weighed and the results were expressed as g decomposed serum/g sample (20).

#### pH and titration acidity measurement

The pH of the yoghurt samples at room temperature was determined using a pH meter (Inolab pH 7,110). 10 mL of pure water was added to 10 g of yoghurt sample and titrated with 0.1 N NaOH until the pH value of the mixture reached 8.1 and titration acidity was calculated according to the consumed 0.1 N NaOH value. Total acidity of yoghurt samples was determined in terms of lactic acid (21).

#### Color analysis

The surface color values (L^*^, a^*^, b^*^) of the yoghurt samples were determined using a HunterLabScan colorimeter (HunterAssociatesLaboratory, Inc., Reston, VA, United States) according to the method described by Britannica (22). The “L^*^” value indicates brightness (whiteness or lightness vs. darkness); the “+a^*^” value signifies red; the “−a^*^” value denotes green; the “+b^*^” value represents yellow, and the “−b^*^” value indicates blue.

#### Texture analysis

The firmness and stickiness of the functional yoghurt were characterized using a Texture Analyzer (Model: TA.XT.2 Plus, Stable Microsystems) and Texture Exponent Software. A Back Extrusion Cell (A/BE) equipped with a 35 mm disk and extension rod, utilizing a 5 kg load cell was employed to assess the firmness and consistency of the yoghurt samples. Five measurements for each sample were recorded using a 5 mm diameter and 150 mm long stainless steel probe adapter connected to a 5 kg load cell. The penetration depth at the geometric center of the specimens in a standard sized back extrusion vessel was set at 30% strain and the penetration speed was set at 1.0 mm/s. The stiffness of the specimens was determined as the peak compression for penetration. The highest negative force was utilized as an indicator of consistency/resistance to flow from the disk during back extrusion.

#### Microbiological analyses

Total aerobic mesophilic bacteria (TMAB), lactic acid bacteria (LAB), yeast-mold and coliform bacteria were quantified in yoghurt samples. 10 g of yoghurt sample was taken and then they were serially diluted in saline solution. TMAB counts were determined on Plate Count Agar (PCA). LAB were enumerated by pour plate method using MRS agar incubated at 30 °C for 72 h (23). Total yeast and mold counts were performed by spread plate method on Yeast Extract Glucose Chloramphenicol Agar (YGC) and agar plates left to incubation at 25 °C for 3–5 days. Pouring plate method was used for analysis of coliform bacteria counts using Violet Red Bile Dextrose (VRBD) agar and aerobic incubation at 37 °C for 1 day. The results were calculated as log cfu/g.

### Effect of *in vitro* digestion on bioactive compounds

*In vitro* digestion method by Brodkorb et al. (24) was used to determine the digestibility of phenolic compounds in yoghurt samples to which proniosomes were added. In this method, the digestion of the samples in the mouth, stomach and intestine was simulated, respectively. In the oral phase digestion model; 5 mL sample was mixed with 5 mL simulated salivary fluid in a 50:50 (v/v) ratio. While preparing the simulated salivary fluid stock solution, 15.1 mM KCl, 13.6 mM NaHCO3, 3.7 mM KH_2_PO_4_, 0.15 mM MgCl_2_(H_2_O)6, 0.06 mM (NH_4_)_2_CO_3_ were added and digested at 37 °C at pH 7.0 for 2 min in a mixture of α-amylase enzyme (75 U/mL) produced from human saliva. After oral phase digestion, gastric phase digestion was started. Samples from the oral phase digestion were mixed with simulated gastric fluid as 50:5 (v/v). While preparing the stock solution of simulated gastric fluid, 6.9 mM KCl, 0.9 mM KH_2_PO_4_, 47.2 mM NaCl, 25 mM NaHCO_3_, 0.1 mM MgCl_2_(H_2_O)6, 0.5 mM (NH_4_)_2_CO_3_ were added and digested with pepsin (2000 U/mL) at pH 3.0 for 2 h by adding 1 M HCl. Ultrapure water was added to dilute the stock solution. In the small intestine digestion model, samples were prepared with simulated intestinal fluid at a ratio of 50:5 (v/v). While preparing the simulated intestinal fluid stock solution, 6.8 mM KCl, 0.8 mM KH_2_PO_4_, 85 mM NaHCO_3_, 38.4 mM NaCl, 0.33 mM MgCl_2_(H_2_O)_6_, 0.5 mM (NH_4_)_2_CO_3_ were added and the pH of the mixture was adjusted to 7, The pH of the mixture was adjusted to 7 and digested with a mixture of enzyme extract (100 U/mL pancreatin and 10 mmol/L bile) and 1 M NaOH for 2 h. After the process, 1 mmol/L AEBSF protease inhibitor was added to the medium and the reaction was terminated.

### Sensory evaluation

Sensory analysis of the yoghurt samples was conducted by a panel of eight trained panelists under approved ethical guidelines. The samples were coded with randomly selected three-digit numbers. Panel members were provided with water to neutralize their palate before the evaluation and between sample assessments. The panel evaluated the samples in terms of color, odor (distinctive pleasant smell), acidity/sourness, cooked taste, oxidized flavor, appearance (uniform color distribution and smooth texture), consistency (visual and superficial consistency, firm texture), texture, taste (distinctive slightly sour taste), and overall acceptability using a hedonic scale ranging from 0 to 9. The analysis included plain yoghurt, yoghurt with free extract, and yoghurt with proniosomal extract.

### Volatile compound analysis

Volatile compounds were identified and quantified after analyzed by GC-FID (7890B, Agilent Technologies). Three grams of yoghurt samples were weighed and placed in a 10 mL vial. Solid phase micro extraction (SPME) was used for the extraction of volatile compounds in the samples. The volatile components formed in the headspace of the samples placed in the vial were injected into the GC/MS. Here, the components in yoghurt are separated according to their volatility and ionized in the mass spectrometer and separated according to the mass/charge ratio (25). Equilibration time, extraction and injection steps were performed by an automated injection module (GC Injector 80; Agilent). The gas chromatography/mass spectrometry (GC/MS) program was set and the vial was kept at 30 °C for 30 min to allow the headspace to reach equilibrium. SPME fiber (Stableflex 2 cm) coated with 75 μm DVB/CAR/PDMS was purchased from Supelco Inc. and used for the analysis. NIST11, Wiley7 and Flavor libraries were used for the identification of compounds (26). Quantification of each flavor compound was performed using 4-nonanol (2 μL) as internal standard. Separation of aroma compounds was carried out using a DB-Wax column (30 m × 250 μm × 0.25 μm, J&W Scientific-Folsom). The column temperature was set at 40 °C for 4 min and then increased by 4 °C per minute to 240 °C. Helium was used as carrier gas. The helium flow rate was set to 1 mL/min (27).

### Statistical analysis

The data obtained in the analyses were given as mean ± standard deviation. For the statistical evaluation of the data, one-way analysis of variance (ANOVA) method was applied using Minitab 17 (Minitab Inc., State College, United States) and the differences between the mean values were evaluated at 95% confidence interval and compared according to Fisher’s least significant difference method.

## Results and discussion

### pH and acidity of yoghurt samples

The pH and acidity values of yoghurt to which proniosomal extract and free extract were added are shown in Table 1a,b. According to the statistical evaluation, there is no significant difference between the pH values of the yoghurt samples to which proniosomal extract was added. However, a statistically significant difference was determined between the total acidity values (*p* < 0.05). There was a statistically significant difference in pH and acidity values of the samples to which free extract was added post fermentation (*p* < 0.05). There was no significant difference between the pH and acidity values of the yoghurt to which proniosomal extract was added pre fermentation. However, a statistically significant difference was determined between the pH and acidity values of yoghurt with free extract (*p* < 0.05). According to the results, it was determined that pH and acidity values of yoghurt containing proniosome and extract did not differ according to the amounts in both productions. Considering the pH values of both productions, the pH values of the yoghurt samples in which proniosomal structure was added pre and post fermentation were lower than the pH values of the yoghurt samples in which extract was added. This situation was interpreted that this may be due to the ingredients used for the formation of the proniosomal powder. In a study, it was reported that the pH and acidity of oleuropein and olive phenolics (tyrosol and hydroxytyrosol) added milk and yoghurt showed similar values in terms of pH and acidity, and the pH values were in the range of 4.30–4.40 (28).

**Table 1:** Physicochemical analysis results of yoghurts with different amounts of proniosomes and extracts.

Samples	pH	Acidity (%)	Syneresis (%)	*L* ^*^	*a* ^*^	*b* ^*^
a)						
C	4.02 ± 0.11	0.33 ± 0.002 ^c^	37.0 ± 0.8^a^	95.5 ± 0.05^e^	−1.92 ± 0.00^a^	6.25 ± 0.01^f^
P01	4.13 ± 0.12	0.34 ± 0.004^bc^	36.0 ± 0.4^ab^	96.2 ± 0.01^c^	−1.95 ± 0.00^d^	6.79 ± 0.01^e^
P02	4.18 ± 0.1	0.33 ± 0.003^bc^	36.1 ± 0.38^ab^	96.4 ± 0.03^a^	−1.94 ± 0.005^d^	6.98 ± 0.00^c^
P03	4.11 ± 0.07	0.34 ± 0.003^a^	35.4 ± 0.63^bc^	96.2 ± 0.04^c^	−1.94 ± 0.00^c^	6.89 ± 0.005^d^
P04	4.11 ± 0.06	0.34 ± 0.003^abc^	34.6 ± 1.01^cd^	96.3 ± 0.05^b^	−1.93 ± 0.005^b^	7.08 ± 0.005^b^
P05	4.17 ± 0.005	0.34 ± 0.004^ab^	33.5 ± 0.88^d^	96.1 ± 0.03^d^	−1.93 ± 0.00^b^	7.27 ± 0.01^a^
C	4.46 ± 0.03^c^	0.33 ± 0.001^c^	41.7 ± 0.72^a^	95.3 ± 0.02^c^	−2.06 ± 0.01	6.8 ± 0.12^d^
E01	4.41 ± 0.02^d^	0.35 ± 0.005^a^	40.1 ± 0.51^b^	95.3 ± 0.09^c^	−2.06 ± 0.02	6.83 ± 0.08^d^
E02	4.52 ± 0.01^a^	0.33 ± 0.00^c^	40.2 ± 0.66^b^	96.0 ± 0.11^a^	−2.06 ± 0.02	7.57 ± 0.08^b^
E03	4.5 ± 0.01^ab^	0.33 ± 0.001^c^	39.6 ± 0.55^b^	95.4 ± 0.1^c^	−2.08 ± 0.01	7.15 ± 0.06^c^
E04	4.48 ± 0.00^bc^	0.34 ± 0.002^b^	40.1 ± 0.27^b^	95.8 ± 0.1^b^	−2.06 ± 0.01	7.72 ± 0.06^a^
E05	4.47 ± 0.01^bc^	0.33 ± 0.001^c^	39.6 ± 0.78^b^	95.7 ± 0.06^b^	−2.06 ± 0.01	7.73 ± 0.05^a^
b)						
C	4.10 ± 0.005	0.41 ± 0.02	34.41 ± 1.27^a^	94.37 ± 0.46	−1.85 ± 0.01^a^	5.65 ± 0.03^f^
P01	4.08 ± 0.02	0.40 ± 0.008	33.30 ± 0.82^a^	94.99 ± 0.35	−1.94 ± 0.005^b^	6.4 ± 0.01^c^
P02	4.09 ± 0.02	0.42 ± 0.01	34.11 ± 0.85^a^	95.20 ± 0.14	−1.94 ± 0.005^b^	7 ± 0.01^a^
P03	4.11 ± 0.005	0.40 ± 0.01	33.72 ± 0.60^a^	94.88 ± 0.48	−1.94 ± 0.005^b^	6.24 ± 0.005^e^
P04	4.09 ± 0.005	0.41 ± 0.006	33.74 ± 0.34^a^	95.19 ± 0.35	−1.94 ± 2.71^b^	6.92 ± 0.02^b^
P05	4.09 ± 0.005	0.39 ± 0.006	31.64 ± 0.55^b^	95.19 ± 0.67	−1.94 ± 0.005^b^	6.34 ± 0.01^d^
C	4.23 ± 0.005	0.53 ± 0.004^c^	30.34 ± 1.40	95.36 ± 0.02^c^	−2.06 ± 0.02	6.8 ± 0.13^d^
E01	4.27 ± 0.03	0.51 ± 0.005^d^	30.08. ± 0.35	95.39 ± 0.1^c^	−2.06 ± 0.03	6.83 ± 0.09^d^
E02	4.22 ± 0.03	0.53 ± 0.001^bc^	29.54 ± 1.17	96.00 ± 0.11^a^	−2.06 ± 0.02	7.57 ± 0.08^b^
E03	4.23 ± 0.07	0.54 ± 0.01^bc^	29.26 ± 0.70	95.45 ± 0.10^c^	−2.08 ± 0.01	7.15 ± 0.06^c^
E04	4.20 ± 0.06	0.5 ± 0.01^b^	29.89 ± 0.56	95.83 ± 0.10^b^	−2.06 ± 0.01	7.72 ± 0.07^a^
E05	4.2 ± 0.06	0.56 ± 0.006^a^	29.06 ± 0.60	95.70 ± 0.06^b^	−2.06 ± 0.01	7.73 ± 0.05^a^

a) Yoghurts with different amounts of proniosomes and extract in post- fermentation and b) yoghurts with different amounts of proniosomes and extract in pre- fermentation. C, Control yoghurt sample; P01, yoghurt containing 0.1 g proniosomes; P02, yoghurt containing 0.2 g proniosomes; P03, yoghurt containing 0.3 g proniosomes; P04, yoghurt containing 0.4 g proniosomes; P05, yoghurt containing 0.5 g proniosomes; E, control yoghurt for extract; E01, yoghurt containing 16 mg free extract; E02, yoghurt containing 33 mg free extract; E03, yoghurt containing 50 mg free extract; E04, yoghurt containing 66 mg free extract; E05, yoghurt containing 83 mg free extract. Samples with added proniosomes and free extracts were statistically evaluated separately. ^a–e^Different letters shown within the columns indicate a statistically significant difference between the samples.

### Syneresis analysis

One of the general quality analyses of yoghurt is syneresis. Prevention of phase separation and reduction of syneresis during storage of yoghurt is one of the main objectives in the dairy industry (29).

Table 1a,b shows the syneresis (%) values of yoghurt to which varying amounts of proniosomal extract and free extract were added post fermentation. As a result of the statistical analysis of syneresis values, it was determined that there was a significant difference between the samples to which proniosome was added post fermentation, while no significant difference was detected between the samples to which extract was added, except for the control sample. The syneresis values of plain yoghurt and yoghurt enriched with varying amounts (0.1 g–0.5 g) of proniosomal extract varied between 33.5 and 37%. As seen in the tables, the syneresis rate of the yoghurt sample decreased with the addition of free extract and nanocapsulated extract compared to the control sample. The difference between the syneresis values of the samples to which proniosome was added post fermentation was statistically significant (*p* < 0.05). Due to the increase in the total dry matter of yoghurt, the water holding capacity increases and the porosity of yoghurt decreases, which increases the stability of the gel network of yoghurt and minimizes the syneresis rate (30). This behavior may be associated not only with increased total solids but also with the structural contribution of the proniosomal system, which may enhance water retention within the gel network. In addition, in this study, the decrease in syneresis in yoghurt with increasing amounts of proniosome was more pronounced. Here, due to the presence of maltodextrin, the absorption of water is accelerated by the addition of proniosome and thus the syneresis process is further delayed. In a study investigating the potential use of waste cinnamon leaves in yoghurt, it was indicated that encapsulation of waste cinnamon leaf extract increased the stability of yoghurt, so that the degree of syneresis of yoghurt to which encapsulated extract was added was at the lowest level (9). Feng et al. (31) investigated the effect of calcium alginate/collagen hydrolysate particles encapsulating high content tea polyphenols (CA/CHBs-e HCTPs) on the quality characteristics of yoghurt during cold storage and the results showed that the addition of CA/CHBs-e HCTPs significantly reduced the water holding capacity of yoghurt compared to control yoghurt and the increase of CA/CHBs-e HCTPs from 0 to 15 (g/100 g) further reduced syneresis.

### Color analysis

In this study, L^*^ values were compared to examine the lightness/whiteness of yoghurt samples by image processing, as high values of L^*^ indicate bright samples. Statistical analyses showed that there was no statistically significant difference in L^*^ values between the control and the yoghurt enriched with proniosomal extract (*p* > 0.05). According to García-Pérez et al. (32), color indices are related to pH and a decrease in pH may lead to a decrease in the L^*^ index of yoghurt. Table 1a,b shows that the L^*^ index and pH values of the yoghurt samples containing proniosome and free extract did not differ from the control sample.

### Texture analysis

The studied textural parameter values (firmness and adhesiveness) of yoghurt with different concentrations of proniosomal OLBE (0.1 g–0.5 g) and equivalent, free form OLBE (16 mg–83 mg) and control (plain) yoghurt post fermentation are summarized in Table 2a,b. Yoghurts to which different concentrations of proniosomal OLBE (0.1 g–0.5 g) and equivalent, free form OLBE (16 mg–83 mg) were added pre fermentation are shown in Table 2c,d. According to the results of texture analysis performed to determine whether there were any textural differences due to the addition of proniosomes and extracts, the firmness and adhesiveness values of the yoghurt containing proniosomes and extracts post fermentation decreased as the proniosome and extract concentrations increased compared to the control yoghurt. In pre-fermentation yoghurt, as the proniosome and extract concentrations increased, an irregular increase and decrease was observed in terms of firmness and adhesiveness compared to control yoghurt. This may be associated with differences in the dissolution behavior of proniosomal and free extracts at higher processing temperatures, as well as the influence of proniosomal structures on protein network formation and interactions within the yoghurt matrix. However, these differences in both post-fermentation and pre-fermentation yoghurt with proniosome and extract additions may not be easily visible in the final product for the consumer. In a previous study for yoghurt enriched with resveratrol-containing niosomes, which obtained similar results to our study, a decrease in texture analysis results was observed in yoghurt with encapsulated niosomes compared to control yoghurt (33).

**Table 2:** Firmness and adhesiveness values of yoghurts samples.

Samples	Firmness (gF)	Adhesiveness (gF)
a)		
C	15.08 ± 0.26^a^	−11.74 ± 0.16^d^
P01	14.88 ± 0.20^ab^	−11.44 ± 0.31^d^
P02	14.52 ± 0.36^ab^	−10.75 ± 0.12^c^
P03	14.22 ± 0.41^b^	−10.82 ± 0.24^c^
P04	12.33 ± 0.47^d^	−8.75 ± 0.19^a^
P05	13.32 ± 0.47^c^	−9.34 ± 0.07^b^
b)		
C	16.59 ± 0.55	−11.99 ± 0.31
E01	17.63 ± 0.51	−12.73 ± 0.53
E02	17.54 ± 0.60	−12.49 ± 0.45
E03	17.74 ± 0.68	−12.94 ± 0.17
E04	16.99 ± 0.60	−12.23 ± 0.32
E05	17.64 ± 0.55	−12.64 ± 0.14
c)		
C	13.26 ± 0.22	−9.76 ± 0.40
P01	13.45 ± 0.49	−4.78 ± 0.49
P02	12.65 ± 1.14	−6.50 ± 5.74
P03	12.99 ± 0.42	−9.50 ± 0.13
P04	13.12 ± 0.22	−9.55 ± 0.13
P05	13.79 ± 0.70	−10.05 ± 0.50
d)		
C	12.89 ± 0.70^b^	−5.44 ± 7.1
E01	9.85 ± 0.72^c^	0.35 ± 8.00
E02	12.29 ± 0.81^b^	−5.47 ± 5.00
E03	13.13 ± 0.23^ab^	−9.23 ± 0.57
E04	12.99 ± 0.36^b^	−9.35 ± 0.27
E05	14.08 ± 0.26^a^	−10.29 ± 0.18

a) Yoghurts with proniosomes added post-fermentation; b) Yoghurts with extract added post-fermentation; c) Yoghurts with proniosomes added pre-fermentation; d) Yoghurts with extract added pre-fermentation. No statistically significant difference was determined (*p* > 0.05). C, control yoghurt sample; P01, yoghurt containing 0.1 g proniosomes; P02, yoghurt containing 0.2 g proniosomes; P03, yoghurt containing 0.3 g proniosomes; P04, yoghurt containing 0.4 g proniosomes; P05, yoghurt containing 0.5 g proniosomes; E, control yoghurt for extract; E01, yoghurt containing 16 mg free extract; E02, yoghurt containing 33 mg free extract; E03, yoghurt containing 50 mg free extract; E04, yoghurt containing 66 mg free extract; E05, yoghurt containing 83 mg free extract.

^a–d^Different letters indicate significant differences (*p* > 0.05).

### Determination of antioxidant capacity

**Figure 1:**
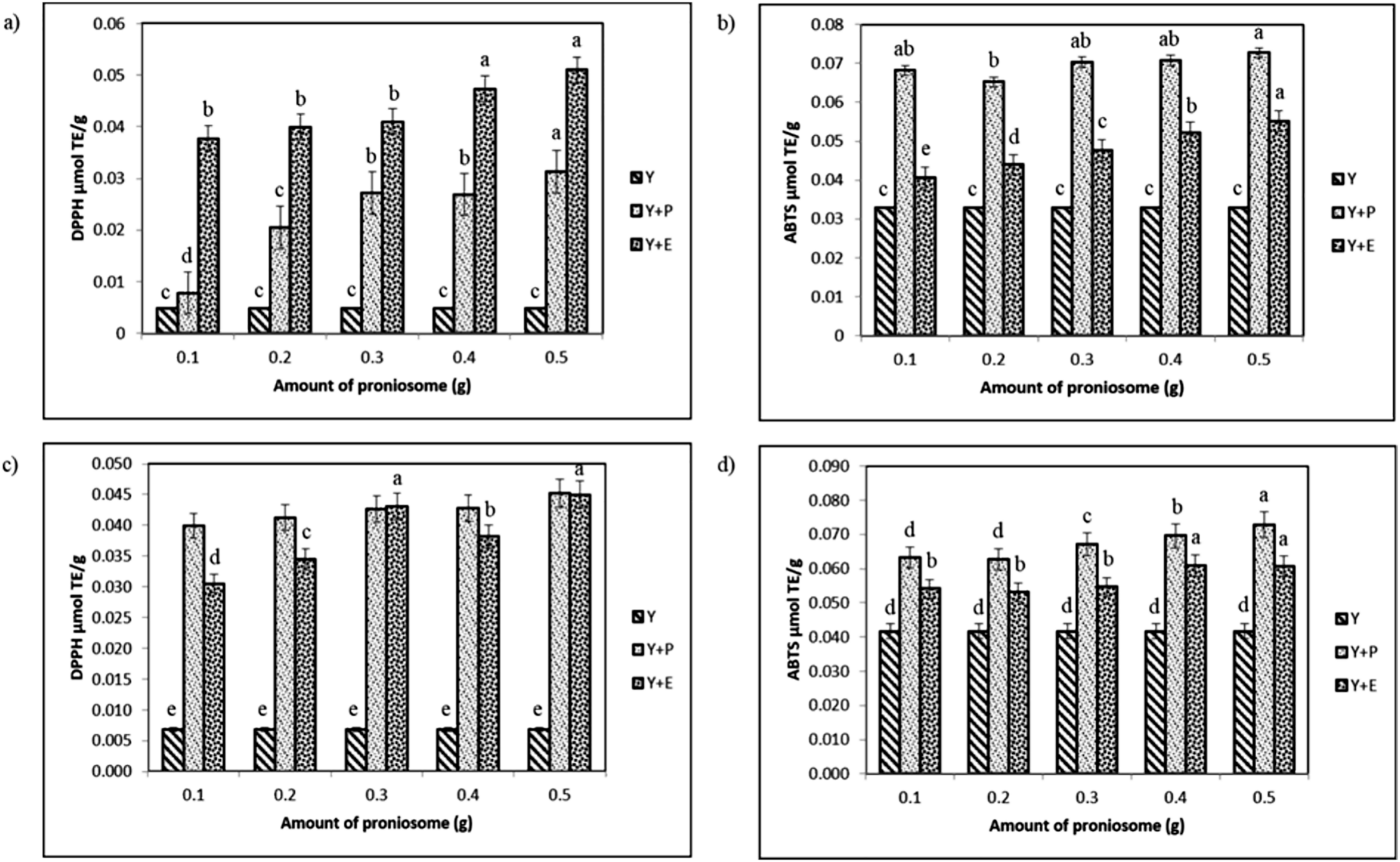
Antioxidant capacity of yoghurt samples in DPPH and ABTS methods: **(a)** Proniosome and extract added post-fermentated yoghurt samples; **(b)** proniosome and extract added post-fermentated yoghurt samples; **(c)** proniosome and extract added pre-fermented yoghurt samples; **(d)** proniosome and extract added pre-fermentated yoghurt samples.

The antioxidant capacities of yoghurts with different amounts of proniosomes added pre and post fermentation were determined using DPPH and ABTS methods. DPPH antioxidant capacity analysis was performed using control yoghurt samples, proniosome-added yoghurt samples, and extract-added yoghurt samples. Statistically, it was determined that the difference between yoghurt samples with proniosomes added pre and post fermentation was significant (*p* < 0.05). The results of DPPH antioxidant capacity analysis post fermentation are given in Figure 1a and ABTS antioxidant capacity analysis results are given in Figure 1b. When analyzing the antioxidant capacity results of yoghurt samples by the ABTS method post fermentation, the antioxidant capacity results of the proniosome-added samples were found to be higher. This may be explained by the ability of the ABTS assay to evaluate both hydrophilic and lipophilic antioxidant systems, which could enhance the detection of encapsulated compounds released from the proniosomal structure. It was determined that there was a statistically significant difference between the proniosome-added samples (*p* < 0.05). The highest antioxidant capacity value was obtained in the yoghurt sample with 0.5 g (P05) proniosome added.

As a result of antioxidant capacity analyses, it was found that there were differences between the DPPH and ABTS methods. While the antioxidant capacity of the yoghurt samples with the extract added was higher in the DPPH method, the antioxidant capacity of the proniosome-added samples was higher in the ABTS analysis. The ABTS analysis is based on the formation of the blue/green ABTS radical and can be applied to lipophilic and hydrophilic antioxidant systems. The DPPH analysis uses radicals dissolved in organic media and therefore represents a more applicable analysis to hydrophobic systems (34). Here, these results suggest that the differences are likely related not only to the analytical methods but also to the interaction of encapsulated compounds with the surrounding matrix.

For pre-fermentation yoghurt samples, the results of the DPPH antioxidant capacity analysis are given in Figure 1c and the ABTS antioxidant capacity analysis results are given in Figure 1d. According to the findings, the antioxidant capacity of the proniosome-added yoghurt samples was higher in both methods. Here, the DPPH antioxidant capacity was found to be higher in yoghurt samples with proniosomes added pre fermentation, as opposed to yoghurt samples with proniosomes added post fermentation. Improved dissolution of the proniosomal formulation at higher processing temperatures is likely to influence the dispersion and distribution of encapsulated compounds within the matrix, thereby affecting their subsequent release and measurable antioxidant activity.

In a study, the efficiency of the ABTS and DPPH methods were compared using antioxidant-rich fruit, vegetable, and beverage samples, and it was stated that the ABTS method better reflected the antioxidant capacity of colored and hydrophilic samples (35). In a study by El-Said et al. (36), it was reported that the addition of encapsulated doum extract phenolics to yoghurt samples resulted in yoghurt with similar characteristics to control yoghurt but with higher antioxidant capacity. In a study evaluating the physicochemical and antioxidant properties of yoghurt enriched with liposomal olive leaf phenolics, it was found that the antioxidant activity of yoghurt samples containing free and encapsulated olive leaf extract was significantly higher than plain yoghurt (17). Yoghurt enriched with olive leaf and extract added pre and post fermentation showed higher antioxidant activity compared to control samples. In addition, the antioxidant activity values of yoghurt samples enriched with olive leaf and extract post fermentation were found to be higher than the samples enriched pre fermentation (37).

In another study, the highest antioxidant activity was determined in yoghurt samples enriched with lyophilized microencapsulated pomegranate peel extract, and the lowest in plain yoghurt (38). Similar to previous studies (17, 37), the antioxidant capacities of yoghurt samples enriched with proniosomal and free form extracts were found to be higher than control (plain) yoghurt in our study. The higher antioxidant activity observed in yoghurt samples containing proniosomal extract compared to the control can be attributed to the controlled release behavior of the proniosomal system, which protects phenolic compounds from degradation and regulates their availability.

### Microbiological analyses

**Figure 2:**
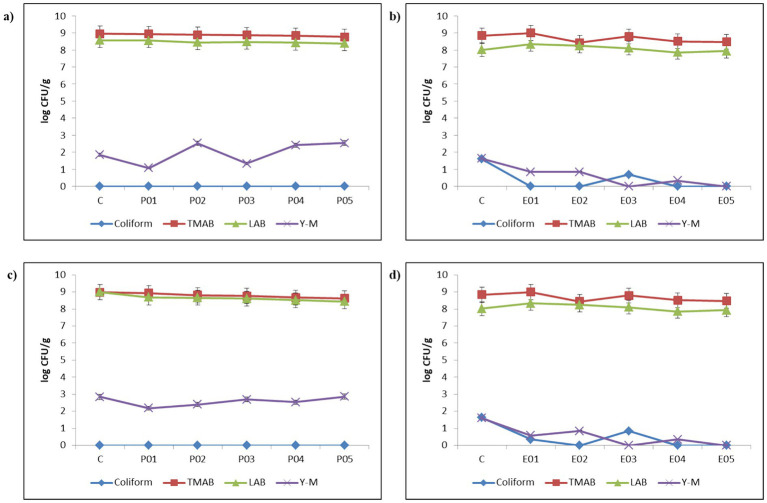
Microbiological count results (log CFU/g) of yoghurts **(a)** with proniosomes added post-fermentation (K-P05), **(b)** with extract added post-fermentation (K-P05), **(c)** with proniosomes added pre-fermentation (K-P05), **(d)** with extract added pre-fermentation (K-P05).

Post-fermentation, microbiological analyses (total mesophilic aerobic bacteria (TMAB), lactic acid bacteria (LAB), yeast-mold and coliform bacteria) were performed on yoghurts with different amounts of proniosome and extract. The results of the counts were expressed as log CFU/g and shown in Figures 2a,b. According to the results, TMAB counts ranged from 8.93–8.76 log CFU/g in yoghurts with proniosome extract added, 8.95 log CFU/g in the control yoghurt sample of this production, 8.99–8.47 log CFU/g in yoghurts with free extract added, and 8.84 log CFU/g in the control yoghurt sample of this production. LAB counts ranged from 8.55–8.37 log CFU/g in yoghurts with proniosome extract added, 8.56 log CFU/g in the control yoghurt sample of this production, and 8.33–7.93 log CFU/g in yoghurts with free extract added, and 8.01 log CFU/g in the control yoghurt sample of this production.

Pre-fermentation, the microbial counts of yoghurts with different amounts of proniosome and extract are shown in Figures 2c,d. According to the results, TMAB counts were between 8.93–8.63 log CFU/g in yoghurts with proniosomal extract, 8.98 log CFU/g in the control yoghurt, 9–8.44 log CFU/g in yoghurts with free extract, and 8.84 log CFU/g in the control yoghurt sample of this production. LAB counts were between 8.68–8.44 log CFU/g in yoghurts with proniosomal extract, 8.99 log CFU/g in the control yoghurt sample, and between 8.33–7.94 log CFU/g in yoghurts with free extract, and 8.02 log CFU/g in the control yoghurt sample of this production.

It was observed that both pre and post fermentation, the total aerobic mesophilic bacteria and lactic acid bacteria counts in yoghurts with different amounts of proniosomes and extracts slightly decreased but did not cause significant changes. Although the initial bacteria counts vary in different types of yoghurt and fermented milk products, the bacteria present in yoghurt are thought to be beneficial to human health. However, this count should not be less than 10^7^ CFU/mL in yoghurt, and *Lactobacillus delbrueckii subsp. bulgaricus* and *Streptococcus thermophilus* should be included among the starter bacteria (39).

In one study, the addition of *Hibiscus sabdariffa* L. flower marmalade to yoghurt was reported to decrease the total aerobic mesophilic bacteria count and lactic acid bacteria count (40). In another study on the enrichment of strawberry yoghurt with microcapsules, the initial lactobacilli counts decreased with the progress of storage (41). Kim et al. (42) found that the number of *Lactobacillus delbrueckii subsp. bulgaricus* decreased during storage in yoghurts with encapsulated and non-encapsulated iron and vitamin C added, and that there was no significant difference between the samples.

As a result, this decrease was associated with changes in pH, sugar, and acidity in the samples, depending on the increasing concentration of proniosomal and free olive leaf brine extract (43).

Yeast and mold counts, on the other hand, ranged from 1.08–2.54 log CFU/g in yoghurts with proniosomal extract added post fermentation, <1–1.62 log CFU/g in yoghurts with free extract added; and pre fermentation, they ranged from 2.18–2.87 log CFU/g in yoghurts with proniosomal extract, and <1–1.60 log CFU/g in yoghurts with free extract.

Yeast and mold counts are considered an important indicator of the shelf life and quality of yoghurt. According to the TS 1330 Yoghurt Standard, a maximum of 100 CFU/g of mold is allowed in commercially sold yoghurt (44). In this context, it can be said that these yoghurts comply with the standard.

According to the coliform bacteria count result conducted to determine the microbiological quality of yoghurts post fermentation, coliform bacteria were not detected in yoghurts with different amounts of proniosomal extract added, and only in the K (1.59) and E03 (0.69) samples among the yoghurts with free extract. According to the coliform bacteria count result of yoghurts pre fermentation, coliform bacteria were not detected in yoghurts with different amounts of proniosomal extract, and were only observed in the K (1.63), E01 (0.35), and E03 (0.85) samples among yoghurts with free extract, but did not exceed the maximum coliform bacteria count allowed in commercially sold yoghurt. This shows that the produced yoghurt samples comply with the standard and are microbiologically safe for human consumption.

### Volatile compounds of yoghurt

Yoghurt is a product made by fermenting milk using a symbiotic culture of lactic acid bacteria. Lactic acid, produced through the fermentation of lactose, denatures milk protein, creating yoghurt’s structure (via formation of a coagulated gel) and its characteristic taste. During this process, small amounts of by-products are also produced, giving specific flavors and aromas (45). In this study, the aroma profiles of yoghurt samples with increasing amounts of proniosome and free OLBE was examined. The examination results showed that post-fermentation yoghurts with proniosome and extract contained a total of 8 carboxylic acids, 4 aromatic hydrocarbons, and 1 ketone aroma component (Table 3); pre-fermentation yoghurts with proniosome and extract contained 8 carboxylic acids, 1 aromatic hydrocarbon, 1 alcohol, 1 ketone, and 1 phenol aroma component (Table 4). The addition of extract and proniosomes to yoghurt samples caused some differences in yoghurt aroma components.

**Table 3:** Volatile compounds of yoghurt samples with proniosomes and extracts added post-fermentation.

No	RT	LRI	Chemical group	Compound	C	P01	P02	P03	P04	P05	E01	E02	E03	E04	E05
					Concentration (μg/kg)										
1	15.468	1236	Aromatic hydrocarbon	Styrene	—	—	—	—	—	—	1.17	3.42	3.08	1.64	1.5
2	20.848	1325	Aromatic hydrocarbon	(1-methylethenyl)-benzene	—	—	—	—	—	—	2.96	4.9	—	—	—
3	20.856	1325	Aromatic hydrocarbon	1-ethenyl-3-methyl-benzene	—	—	—	—	—	—	—	—	5.01	2.32	3.07
4	26.028	1401	Carboxylic acid	Acetic acid	—	2.83	3.66	3.47	4.63	3.35	—	—	2.49	2.57	3.81
5	26.053	1462	Aromatic hydrocarbon	1-ethenyl-4-ethyl-benzene	77.95	—	—	—	—	—	16.72	17.57	—	—	—
6	32.556	1596	Carboxylic acid	Butanoic acid	4.07	2.89	4.75	1.28	1.41	1.09	0.86	1.14	1.91	1.39	2.16
7	40.094	1800	Carboxylic acid	Hexanoic acid	10.27	7.58	11.88	3.57	3.56	3.39	1.91	2.52	4.39	3.35	4.91
8	42.904	2095	Ketone	4-phenyl-3-butene-2-one	15.8	—	—	—	—	—	—	—	—	—	—
9	44.747	2011	Carboxylic acid	Caprylic acid	7.46	7.67	69.62	4.15	3.31	4.05	1.27	1.72	2.41	2.1	4.65
10	46.260	2128	Carboxylic acid	Nonanoic acid	2.87	—	—	—	—	—	0.79	—	—	—	—
11	47.557	2231	Carboxylic acid	n-Decanoic acid	0.86	3.43	9.32	2.21	1.96	2.13	—	1.07	0.68	0.77	1.73
12	49.088	2387	Carboxylic acid	Benzoic acid	10.34	5.55	9.8	3.72	3.54	3.11	1.69	3	1.4	1.51	2.85
13	49.786	2451	Carboxylic acid	Dodecanoic acid	—	—	—	—	1.05	1.13	—	—	—	—	—

C, control yoghurt sample; P01, yoghurt containing 0.1 g proniosomes; P02, yoghurt containing 0.2 g proniosomes; P03, yoghurt containing 0.3 g proniosomes; P04, yoghurt containing 0.4 g proniosomes; P05, yoghurt containing 0.5 g proniosomes; E, control yoghurt for extract; E01, yoghurt containing 16 mg free extract; E02, yoghurt containing 33 mg free extract; E03, yoghurt containing 50 mg free extract; E04, yoghurt containing 66 mg free extract; E05, yoghurt containing 83 mg free extract.

**Table 4:** Volatile compounds of yoghurt samples with proniosomes and extracts added pre-fermentation.

No	RT	LRI	Chemical group	Compound	C	P01	P02	P03	P04	P05	E01	E02	E03	E04	E05
					Concentration (μg/kg)										
1	26.028	1401	Carboxylic acid	Acetic acid	—	2.08	16	1.65	1.28	1.23	0.34	0.06	—	0.32	—
2	26.053	1462	Aromatic hydrocarbon	1-ethenyl-4-ethyl-benzene	77.95	—	—	—	—	—	—	—	—	—	—
3	32.556	1596	Carboxylic acid	Butanoic acid	4.07	0.74	2.54	—	0.95	0.95	0.72	0.70	1.38	0.87	3.89
4	40.094	1800	Carboxylic acid	Hexanoic acid	10.27	2.94	—	3.0	3.35	3.27	2.76	4.35	4.81	4.27	15.8
5	41.875	1856	Alcohol	Phenethyl alcohol	—	—	—	—	—	—	—	—	—	—	5.50
6	42.904	2095	Ketone	4-phenyl-3-butene-2-one	15.8	—	—	—	—	—	—	—	—	—	—
7	44.747	2011	Carboxylic acid	Caprylic acid	7.46	3.08	17.1	6.45	5.18	4.19	2.79	5.12	4.95	3.77	24.5
8	46.260	2128	Carboxylic acid	Nonanoic acid	2.87	—	—	—	—	—	0.16	1.59	0.06	—	—
9	47.557	2231	Carboxylic acid	*n*-Decanoic acid	0.86	2.25	14.9	—	4.57	3.55	1.42	3.04	3.41	2.18	13.3
10	47.936	2277	Phenol	2,4-2 Tertiary butyl alcohol	—	—	—	—	—	—	—	—	—	0.47	—
11	49.088	2387	Carboxylic acid	Benzoic acid	10.34	2.47	15.3	7.57	4.31	4.29	1.38	1.26	3.21	2.32	10
12	49.786	2451	Carboxylic acid	Dodecanoic acid	—	0.99	—	—	—	2.17	—	—	—	—	—

C, control yoghurt sample; P01, yoghurt containing 0.1 g proniosomes; P02, yoghurt containing 0.2 g proniosomes; P03, yoghurt containing 0.3 g proniosomes; P04, yoghurt containing 0.4 g proniosomes; P05, yoghurt containing 0.5 g proniosomes; E, control yoghurt for extract; E01, yoghurt containing 16 mg free extract; E02, yoghurt containing 33 mg free extract; E03, yoghurt containing 50 mg free extract; E04, yoghurt containing 66 mg free extract; E05, yoghurt containing 83 mg free extract.

When the aroma components of post-fermentation yoghurts with extract and proniosome were examined, styrene, 1-methylethenyl-benzene and 1-ethenyl-3-methyl-benzene aromatic hydrocarbons were detected in control and proniosome-containing yoghurts but not in extract-containing samples. In pre-fermentation proniosome-added yoghurts, phenethyl alcohol and 2,4-2 tertiary butyl alcohol were not detected in control and proniosome-containing yoghurts but were found in extract-containing samples. This suggests that the detection of these components in yoghurts was due to the addition of the extract. Additionally, 1-ethenyl-4-ethyl-benzene and 4-phenyl-3-buten-2-one compounds were found in high amounts in plain yoghurt and decreased or disappeared in proniosome and extract-added yoghurts. Moreover, acetic acid, hexanoic acid, and octanoic acid (caprylic acid), important for yoghurt aroma, were found in higher amounts in both post- and pre-fermentation proniosome and extract-added samples compared to other compounds.

The basic taste of dairy products is mainly derived from natural volatile components in cow’s milk, affected by pasteurization, fermentation, processing, and storage. Most volatile organic compounds present in yoghurt are not produced by starter bacteria but originate from milk (46). Milk is a highly complex food composed of lipids, proteins, carbohydrates, and minerals, and it has been stated that more than 400 volatile compounds are found in dairy products (45).

The flavor-active compounds in yoghurt differ from those in milk, likely due to the metabolism of acid-producing bacteria. These culture-derived aroma compounds result from the microbial, enzymatic, or chemical transformations of lactose, lipids, citric acid, and proteins/amino acids present in milk. The aroma compounds found in yoghurt can basically be divided into four categories: volatile acids (e.g., acetic, propionic, and butyric), non-volatile acids (e.g., lactic, pyruvic, oxalic, and succinic), carbonyl compounds (e.g., acetaldehyde, acetone, acetoin, and diacetyl), and various compounds (e.g., specific amino acids and/or components formed from the thermal degradation of protein, fat, and lactose) (45).

The presence of acetaldehyde, the principal volatile constituent of yoghurt, was not detected in the yoghurts produced in this study. The enzyme alcohol dehydrogenase may reduce acetaldehyde to ethanol. A study reported that the acetaldehyde content in yoghurts generally decreased until 14 days of storage and then showed a significant increase until reaching maximum concentrations at 21 days, followed by a decrease again (47). Study have shown that acetaldehyde concentration decreased during storage, peaked at 14 days, and then decreased again in all evaluated yoghurts (47). These results also support that yoghurt bacteria reduce acetaldehyde through dehydrogenase activity.

During fermentation, different acids are formed in yoghurt through both lipolytic activity and bacterial fermentation. Acetic acid is an important compound produced by lactic acid bacteria fermenting lactose (48). In Cheng’s (45) review, the most common organic acids found in yoghurts are listed as acetic acid, hexanoic acid, and octanoic acid. These acids were generally detected in the yoghurts in this study as well.

### *In vitro* digestive analysis

Phenolic compounds are the primary plant-based antioxidants. Therefore, measuring the total phenolic compounds and their antioxidant activities when added to a food system is essential to understand their function as free radical scavengers and their contributions to human health. Antioxidant potential is determined by various chemical mechanisms such as single electron transfer, hydrogen atom transfer capacity, and metal ion chelation (49). Hence, it’s difficult to determine the antioxidant potential based on a single method. Various antioxidant assays, including DPPH, ABTS, and other total antioxidant capacity (TAC) methods, are widely used to evaluate the antioxidant properties of phenolic compounds Apak et al. (50).

**Figure 3:**
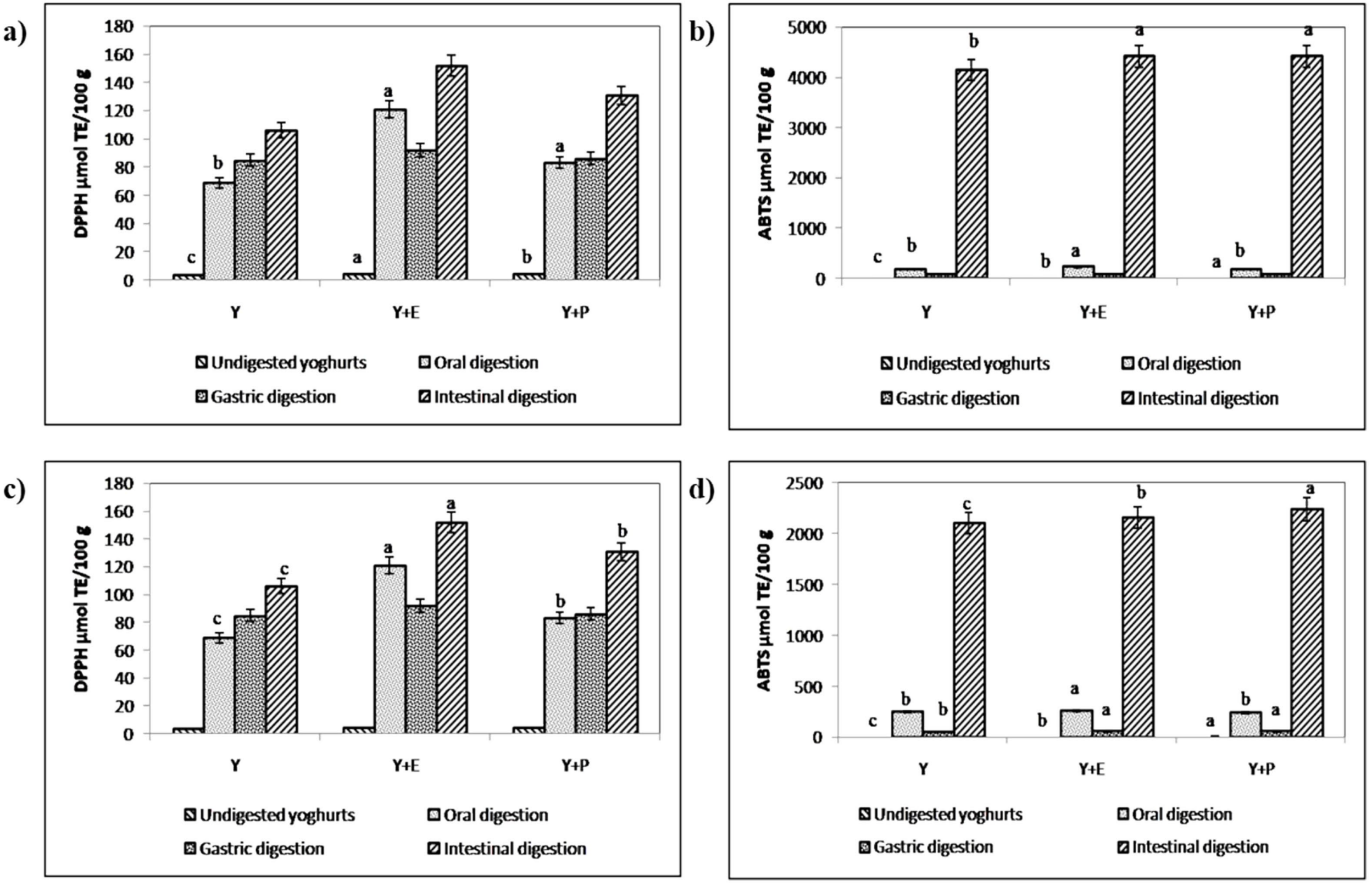
Effect of *in vitro* digestion on the antioxidant activity determined by the **(a)** DPPH method in control yoghurt (Y), proniosome-added yoghurt (Y + P), and extract-added yoghurt (Y + E) samples of post-fermentation **(b)** ABTS method in control yoghurt (Y), proniosome-added yoghurt (Y + P), and extract-added yoghurt (Y + E) samples post-fermentation **(c)** DPPH method in control yoghurt (Y), proniosome-added yoghurt (Y + P), and extract-added yoghurt (Y + E) samples pre-fermentation **(d)** ABTS method in control yoghurt (Y), proniosome-added yoghurt (Y + P), and extract-added yoghurt (Y + E) samples of pre-fermentation.

The effect of encapsulated and free OLBE supplementation on the digestibility of yoghurt was examined by determining the DPPH and ABTS values of yoghurtpre and postfermentation after simulated *in vitro* digestion. As illustrated in Figure 3, the impact of OLBE supplementation and encapsulation on the antioxidant capacities of yoghurts is demonstrated. These capacities were determined using DPPH and ABTS assays in a simulated in vitro digestion system, providing insight into how the encapsulated compounds behave during digestion. Proniosomal encapsulation appears to play a key role in protecting bioactive compounds during gastric digestion and facilitating their release in the intestinal phase, thereby improving their potential bioaccessibility.

The results in Figure 3a prove that enriching yoghurt with OLBE significantly increases its antioxidant activity as determined by the DPPH method. Before digestion, the antioxidant activity determined by the DPPH method in yoghurt with OLBE (17.45 μmol TE/100 g) increased 11.8 times after OLBE supplementation, while the DPPH (13.5 μmol TE/100 g) result with encapsulated OLBE supplementation increased 16.9 times. The release of polyphenols increased during digestion and was found more in the intestinal phase compared to the gastric phase, revealing the protective effect of the proniosomal encapsulation technique against digestive enzymes and pH changes during gastric digestion. The materials used for vesicle bilayer and proniosome stabilization determine the sensitivity of polyphenols to digestive enzymes and pH at each stage (51). Similarly, other studies have shown that both encapsulated blueberry extract (52) and carob pulp extract (53) exhibited an increase in FRAP (Ferric reducing ability of plasma) mostly evident in the intestinal phase during digestion.

During *in vitro* digestion, yoghurt with free extract showed a 7.4% decrease in antioxidant capacity value determined by DPPH. The observed decrease in antioxidant capacity values during gastric and intestinal digestion is expected. When control yoghurt and OLBE-added yoghurt samples are exposed to very low pH in gastric fluid (3.0), followed by exposure to bile salts and enzymes (pancreatin) in the small intestine, it’s reported that these samples hydrolyze and release the remaining phenolic compounds, which are less than the initial amounts (10). However, the increase in antioxidant activity determined by DPPH in yoghurts with proniosomes has been interpreted as possibly related to the release of complex bioactive compounds as a result of the digestion process (54). This finding suggests that different stages of digestion have a significant impact on bioactive molecules, and the antioxidant capacity content of yoghurt was significantly improved by enrichment with proniosomal olive leaf brine extract.

The highest antioxidant activity observed in proniosomal OLBE-added yoghurt samples during the intestinal phase (Figure 3b) can be explained by the degradation of the nanocapsule structure at neutral pH, which promotes the release of encapsulated phenolic compounds. This finding further supports the role of the proniosomal system in enhancing the availability of bioactive compounds during digestion.

Weak activities recorded in the oral and gastric stages of digestion could be due to the limited release of polyphenols from the nanocapsule surface and/or the penetration of nanocapsules because of the presence of surface pores in the oral and gastric fluids (55). However, the differences in antioxidant activities determined by the DPPH and ABTS methods during the digestive stages may not be due to the content of phenolics and anthocyanins, but rather to the variety and properties of the polyphenols present. Additionally, the differences might be related to changes in polyphenol availability associated with the *in vitro* digestion conditions used and/or the release of matrix-associated compounds (56). Similarly, another study applying encapsulated tamarillo polyphenols to yoghurt showed differences in FRAP and CUPRAC activities during the digestive stages of the samples (55). Along with enzymatic action, the effect of pH in oral-gastric-intestinal digestion and the presence of compounds such as peptides or complex polyphenols increase antioxidant activity (54).

Figures 3c,d show the effect of simulated *in vitro* digestion on the antioxidant activity changes determined by DPPH and ABTS methods in yoghurt samples with OLBE and proniosomes added pre-fermentation. Accordingly, in post-fermentation yoghurt samples, the antioxidant activity determined by the DPPH method in the gastric and intestinal phases was found to be significantly higher than those with pre-fermentation additions. The same situation was observed with the ABTS method, with the antioxidant activity determined by the ABTS method in the intestinal phase of post-fermentation yoghurt samples being significantly higher than those with pre-fermentation additions.

The antioxidant capacity values of undigested yoghurt samples were found to be similar in both productions. Post-fermentation antioxidant activity values determined by the DPPH method were observed as 2.8 μmol TE/100 g in control yoghurt, 3.1 μmol TE/100 g in yoghurt with proniosomes, and 5.1 μmol TE/100 g in yoghurt with extract. Pre-fermentation values were found as 3.9 μmol TE/100 g in control yoghurt, 4.5 μmol TE/100 g in yoghurt with proniosomes, and 4.5 μmol TE/100 g in yoghurt with extract.

As a result, it was shown that adding OLBE to yoghurt led to an increase in antioxidant activities determined by both DPPH and ABTS after digestion. Overall, these findings indicate that proniosomal encapsulation not only protects phenolic compounds during digestion but also enhances their bioaccessibility through controlled release mechanisms.

### Sensory analysis of yoghurt samples

**Figure 4:**
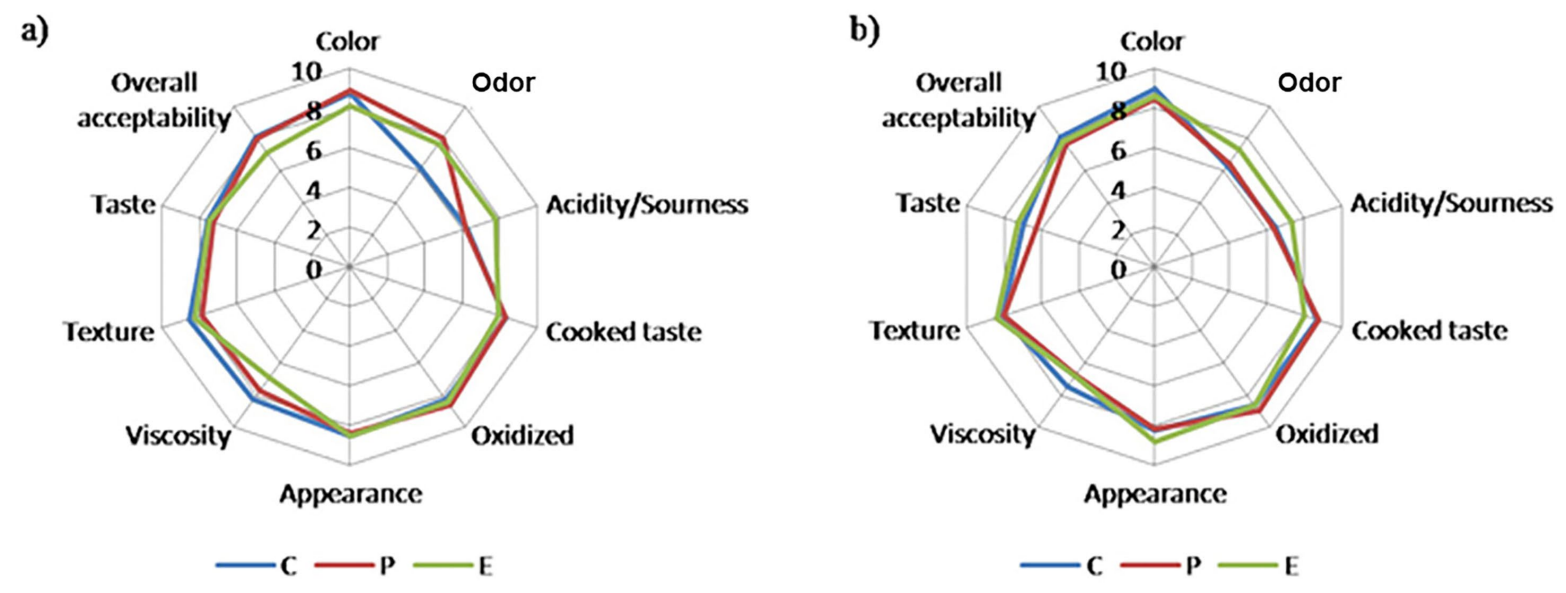
Schematic representation of sensory analysis values **(a)** for yogurt samples with proniosomes and extract added post-fermentation **(b)** for yogurt samples with proniosomes and extract added pre-fermentation. C, Control yogurt; P, Proniosome-containing yogurt; E, Extract-containing yogurt.

The sensory analysis values of yoghurt with proniosomal OLBE and free-form OLBE added post-fermentation, control (plain) yoghurt, and yoghurt with proniosomal OLBE and free-form OLBE added pre-fermentation, control (plain) yoghurt were evaluated for color, odor, acidity/sourness, cooked taste, oxidized taste, appearance, consistency, texture, taste, and overall impression. These are presented as radar charts in Figures 4a,b.

In the sensory evaluation results of yoghurt samples containing 0.5 g of proniosomal extract and equivalent free extract (83 mg) selected from different concentrations (0.1 g–0.5 g), there was no significant difference in sensory parameters among the samples in both post- and pre-fermentation yoghurts. Similar evaluations were made when comparing control yoghurt samples with proniosomal OLBE and free OLBE-added yoghurts. For example, taste values in post-fermentation yoghurts ranged from 7.2 to 7.5; in pre-fermentation yoghurts, taste values ranged from 7 to 7.82. Based on these results, yoghurts with proniosome could be considered preferable functional products.

Thakur et al. (38) subjected yoghurts containing lyophilized microencapsulated pomegranate peel extract at different concentrations (0.5–6%) to sensory analysis using color, texture, taste, aroma, and overall acceptability parameters, and observed that yoghurt enriched at 2% was the most acceptable in all sensory quality characteristics and yielded similar results to the control sample. In another study, sensory evaluation results of flavored yoghurt enriched with microencapsulated *Melissa officinalis* essential oil showed that panelists could not distinguish between samples containing microcapsules; this suggested that the produced *M. officinalis* oil microcapsules could be quickly detected in the mouth after ingestion (57). This model is also consistent with the results of Abedi et al. (58), where yoghurt was enriched with microcapsules of *Nigella sativa* seed oil. In this context, the results showed that adding microcapsules to yoghurt did not significantly affect overall acceptability compared to plain samples.

## Conclusion

This study investigated the comparative effects of proniosomal hydroxytyrosol-rich olive leaf brine extract on the physicochemical, antioxidant, microbiological, textural, sensory, aroma, and *in vitro* digestion properties of yoghurt produced by pre- and post-fermentation addition. The results demonstrated that the incorporation of proniosomal systems is associated with enhanced antioxidant activity and reduced syneresis, without negatively affecting the overall quality attributes of yoghurt. These effects are likely related to the ability of proniosomal structures to protect bioactive compounds and regulate their release within the yoghurt matrix and during digestion. In particular, the improved antioxidant activity observed after in vitro digestion suggests that proniosomal encapsulation enhances the availability of phenolic compounds under gastrointestinal conditions. Based on these findings, post-fermentation incorporation appears to be a more effective approach, offering advantages in terms of processing, as well as protection and controlled release of bioactive compounds in OLBE. Overall, the use of proniosome-based nanostructures not only supports the valorisation of olive leaf by-products but also represents a promising strategy for the development of functional yoghurt with improved bioactive properties.

## Data Availability

The original contributions presented in the study are included in the article/supplementary material, further inquiries can be directed to the corresponding author.
